# Dissolution of Spent Lithium‐Ion Battery Cathode Materials: Overlooked Significance of Aluminum Impurities

**DOI:** 10.1002/advs.202417737

**Published:** 2025-02-28

**Authors:** Kang Liu, Yuying Zhang, Mengmeng Wang, Xiaohong Zhu, Roya Maboudian, Daniel C.W. Tsang

**Affiliations:** ^1^ Department of Civil and Environmental Engineering The Hong Kong University of Science and Technology Clear Water Bay Hong Kong 999077 China; ^2^ Department of Civil and Environmental Engineering University of California Berkeley Berkeley CA 94720 USA; ^3^ Department of Chemical and Biomolecular Engineering University of California Berkeley Berkeley CA 94720 USA

**Keywords:** aluminum interactions, critical mineral extraction, electric vehicle battery, hydrometallurgical dissolution, metal‐oxygen bonding, sustainable waste management

## Abstract

Impurities are pivotal in determining the quality of the critical metal products derived from spent lithium‐ion batteries (LIBs), but there is a lack of understanding of their potential impacts. The mechanisms by which Aluminium (Al) impurities permeate the active crystals of spent ternary nickel─cobalt─manganese oxide (NCM) cathode materials and interact with critical metal sites are elucidated. During frictional contact, the substitution of transition metals by Al impurities disrupts the bonding between critical metals and oxygen, leading to the formation of more stable Al─O covalent bonds. Al can preferentially substitute for Co, altering the local coordination and electronic structure of transition metals. Owing to the strong interaction of Al─O, the Fermi level shifts downward, thus increasing the stabilization of lattice oxygen in the NCM cathode materials and consequently delaying the dissolution of NCM cathode materials. In different extraction environments, the invasion of Al retards the release of lattice oxygen and inhibits the dissolution of NCM in formic acid but enhances those in ammonia solution and shows mixed results in deep eutectic solvents. These crucial findings will help to elucidate the mechanisms of Al impurities in the recycling industry chain of retired LIBs, thereby enhancing the recovery of high‐quality critical metal products.

## Graphical abstract

During the mechanical crushing of retired lithium‐ion batteries, aluminum foil from current collectors may penetrate NCM active crystals through frictional contact, forming more stable Al─O bonds. The dissolution behavior of critical metals in NCM cathodes will be influenced to varying extents by different mechanisms in various hydrometallurgical systems as verified in this study.

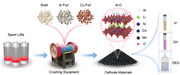

## 1 Introduction

The global electric vehicle industry is experiencing rapid growth attributable to the increasing demand for green and low‐carbon solutions to climate change.^[^ ^1^, ^2^ ^]^ According to the International Energy Agency (IEA) report, global sales of electric vehicles exceeded 14 million units in 2021.^[^ ^3^ ^]^ The quantity of discarded components of these vehicles, such as power lithium‐ion batteries (LIBs), has increased notably.^[^ ^4^, ^5^ ^]^ According to SNE Research's forecast, based on metal content, it is estimated that the global LIB recycling (including waste batteries and materials) market size will be 786 000 tons by 2025.^[^ ^6^ ^]^ Spent LIB cathode materials are composed of technology‐critical metals, such as lithium (Li), nickel (Ni), cobalt (Co), and manganese (Mn).^[^ ^7^, ^8^ ^]^ These secondary materials can be extracted for recycling or directly repurposed to create new electrode materials.^[^ ^9^, ^10^ ^]^ High‐purity separation of technology‐critical metals presents a key industrial challenge to the sustainable supply of LIBs.^[^ ^11^, ^12^ ^]^ The main theme of the global circular economy has also driven the rapid flow of this type of solid waste.^[^ ^13^, ^14^, ^15^ ^]^

The recycling of retired LIBs not only reduces resource waste but also has significant environmental and circular economic benefits.^[^ ^16^, ^17^ ^]^ Common recycling strategies for critical metals in spent LIBs encompass hydrometallurgical processing,^[^ ^18^, ^19^ ^]^ pyrometallurgical refining,^[^ ^20^, ^21^ ^]^ and material regeneration.^[^ ^22^, ^23^ ^]^ To accomplish these steps, disassembling the robust shell and precisely assembling the battery pack is essential.^[^ ^24^ ^]^ The recycling industry relies on mechanically assisted methods for disassembling battery packs, unavoidably leading to the presence of remaining impurities such as aluminum (Al), copper, fluorine, and phosphorus.^[^ ^25^ ^]^ Such impurities potentially pose challenges to the separation and circularity of technology‐critical metals.^[^ ^26^, ^27^ ^]^

Fluorine and phosphorus can convert the Li portion in the cathode material into slightly soluble lithium fluoride and lithium phosphate,^[^ ^28^, ^29^ ^]^ Al foil is most likely to enter the sorted cathode material powder as impurities due to its easy pulverization.^[^ ^30^ ^]^ When the calcination temperature surpasses 500 °C, Li and Al in the cathode material may produce insoluble lithium aluminate crystal.^[^ ^31^, ^32^ ^]^ The Al impurities also affect the progress of electrochemical reactions and reduce the discharge capacity of regenerated LIBs.^[^ ^33^, ^34^, ^35^ ^]^ However, the industry lacks thorough knowledge of the significant roles of Al intrusion into spent LIB cathode material crystals during the extraction of technology‐critical metals.

Herein, we report on the response mechanisms of Al impurities on the dissolution behavior of spent LIB ternary nickel─cobalt─manganese (NCM) cathode materials under various liquid‐phase environments. Although the acidity induced by Al and the reducing capability of metallic Al can accelerate the dissolution of NCM active crystals in the hydrometallurgical system, the combination of Al and NCM active crystals at the early stage of battery recycling may enhance the stability of NCM crystals. This study will elucidate the inhibitory or accelerating effects of Al on the initial dissolution stage of NCM active crystals in hydrometallurgical processes from a microscopic perspective. We demonstrate the process of Al impurities permeating the active crystals of the NCM cathode materials and analyze the interactions between Al and critical metal sites. Employing aberration‐corrected, high‐angle annular dark field scanning transmission microscopy (AC‐HAADF‐STEM), X‐ray absorption near edge structure (XANES), and density functional theory (DFT), we elucidate the mechanisms by which aluminum may interfere with the dissolution of active crystals in NCM cathode materials during the mechanochemical‐metallurgical recycling processes. These crucial findings can help us reveal the potential roles and underlying mechanisms of Al impurities in the recycling chain of retired LIBs, thereby enhancing the recovery of high‐quality critical metal products for resource circularity.

## 2 Results and Discussion

### 2.1 Permeation of Al Impurities into NCM Crystals

**Figure 1:**
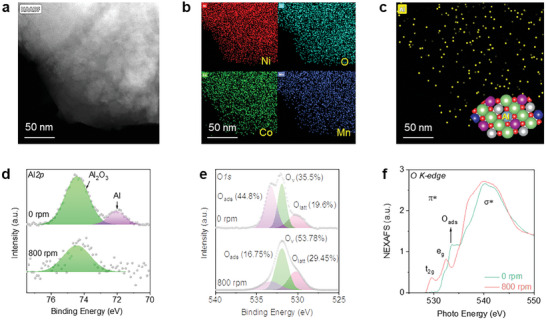
Characterization of Al permeation into NCM Phase. a) AC HAADF‐STEM image (50 nm scale bar). b) EDS mapping images of Ni, Co, Mn, and O (50 nm scale bar). c) EDS mapping image of Al (50 nm scale bar). High‐resolution XPS results of d) Al*2p* and e) O*1s*. f) O K‐edge XANES spectra results. 0 rpm is the sample before, and 800 rpm is the sample after the frictional contact between NCM and Al powders, respectively. XPS, X‐ray photoelectron spectroscopy.

The X‐ray diffaction (XRD) patterns of NCM‐Al samples before and after the frictional contact were compared (Note S1 and Figure S1, Supporting Information). Prior to fictional contact (0‐rpm), the XRD pattern indicates a mixture of NCM and Al. The diffraction peaks of Al (111) and Al (200) gradually become less intensive in the 800‐rpm frictional contact sample, indicating that the interactions between Al powder and NCM occurred. The SEM with EDS mapping, laser particle size analysis (LPSA), high‐resolution transmission electron microscopy, and energy dispersive spectroscopy (HRTEM‐EDS) results are shown in Figures S2–S4 (Supporting Information), respectively. **Figure** **1**a shows an AC HAADF‐STEM image of the NCM‐Al after frictional contact, with the white areas indicating the edge of the morphology of the NCM powder sample. In the EDS mapping analysis, Ni, Co, Mn, and O were identified as the predominant elements (Figure 1b). Furthermore, the permeation of Al into the NCM lattice was confirmed, exhibiting an irregular, punctate scattering distribution (Figure 1c).

Surface species of the samples before (0 rpm) and after (800 rpm) frictional contact between NCM and Al powders were examined using high‐resolution X‐ray photoelectron spectroscopy (XPS) (Figure 1d,e). Characteristic peaks of Al_2_O_3_ and Al were detected on the surface of the NCM‐Al sample at 0 rpm (Figure 1d). After 800 rpm, the characteristic peak of Al disappeared, there remained only the characteristic peak of Al_2_O_3_, confirming the chemical binding of Al with O within the NCM lattice. High‐resolution XPS results of O*1s* (Figure 1e) indicate the convolution peaks of three oxygen species, lattice oxygen (O_latt_), defect oxygen (O_v_), and adsorbed oxygen (O_ads_). After the frictional contact between NCM and Al powders, the proportion of defect oxygen increased significantly from 35.5% to 53.8%. To further elucidate the local environment and coordination changes of O species, XANES analysis was conducted (Figure 1f). The edge front peak at 534.4 eV in the NCM‐Al‐0 sample is attributed to adsorbed oxygen (O_ads_), which is likely caused by radiation damage from the incident beam.^[^ ^36^ ^]^ The σ^*^ peak at 542.6 eV is interpreted as a weak peak formed by the O *1s*‐*2p* transition and high energy multiple scattering. The *π*‐peak intensity in the NCM‐Al‐800 sample increased following frictional contact, possibly due to the anti‐bonding state caused by the intense mixing of O *2p* state and Al *3s* and *3p* states, showing an improvement in the degree of metal‐oxygen hybridization. For the NCM‐Al‐800 sample, the two peaks (π^*^) at 529 and 532 eV correspond to transitions from O *1s* to *t_2g_* and *e_g_*, respectively, probably caused by the hybridization of Al *3p* and O *2p*. The above results confirm that after the frictional contact, Al permeates the lattice of NCM and binds with O.^[^ ^37^ ^]^

### 2.2 Interactions Between Al and Transition Metals in NCM Crystals

**Figure 2:**
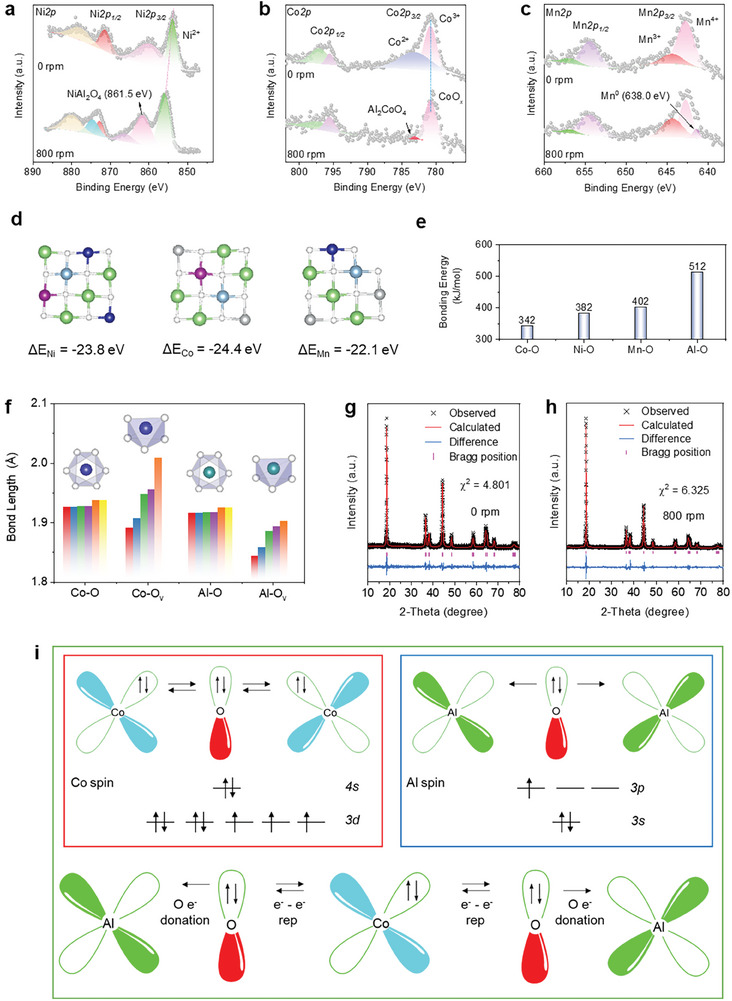
Interactions between Al and transition metals. High‐resolution XPS results of NCM‐Al sample before (0 rpm) and after frictional contact (800 rpm): a) Ni*2p*, b) Co*2p*, c) Mn*2p*. d) formation energy of Al replacing Ni, Co, and Mn atoms (green = Li atoms, white = O atoms, gray = Ni atoms, blue = Co atoms, purple = Mn atoms, and light blue = Al atoms). e) bond energies of Co─O, Ni─O, Mn─O, and Al─O bonds. f) bond lengths of Co─O, Co─O_v_, Al─O, and Al─O_v_. XRD refinement patterns of NCM‐Al samples g) before and h) after frictional contact, and i) crystal field calculation diagram of Co─O─Al bond.

The trends in binding energies of Li and Me (Me═Ni, Co, and Mn) species within Al and NCM crystals were investigated using Gibbs free energy calculations (Figure S5, Supporting Information), thermodynamic phase diagrams (Figures S6 and S7, Supporting Information), high‐solution XPS analysis of Li *1s* (Figure S8, Supporting Information), and FT‐IR spectroscopy (Figure S9, Supporting Information). The near‐surface species compositions of Ni, Co, and Mn elements were examined using high‐resolution XPS spectra. The characteristic peak of NiAl_2_O_4_ (861.5 eV) was observed in the NCM‐Al‐800 sample, confirming the permeation effect of Al on the Ni─O bond (**Figure** **2**a).^[^ ^38^, ^39^ ^]^ The XPS examination of Co *2p* exhibited split characteristic peaks for Co *2p*1/2 and Co *2p*3/2 (Figure 2b). In the 800‐rpm sample, the characteristic peak of Co^2+^ disappeared, and only the new species Al_2_CoO_4_ (783.1 eV) was observed, confirming the chemical binding between Al and Co. In the high‐resolution XPS of Mn*2p*, the Mn*2p*3/2 characteristic peak was split into Mn^4+^ and Mn^3+^ internal peaks for the NCM‐Al‐0 sample. In comparison, after frictional contact at 800 rpm, the Mn^0^ species (638.0 eV) appeared in the Mn*2p*3/2 characteristic peak, confirming the surface reduction of Mn^4+^ and Mn^3+^ by Al permeation (Figure 2c).

DFT calculations were performed to analyze the interactions of Al with Ni, Co, and Mn (Figure 2d). The formation energies of Al permeation to replace the three types of atoms were first calculated, showing that the substitution energy of Al for Co atoms is ΔE_Co_ = ‐24.4 eV, while ΔE_Ni_ and ΔE_Mn_ for Ni and Mn are −23.8 and −22.1 eV, respectively, indicating a higher likelihood of Al substituting for Co, assuming no barriers/constraints in terms of kinetics and morphology. Compared with the Co─O (342 kJ mol^−1^), Ni─O (382 kJ mol^−1^), and Mn─O bonds (402 kJ mol^−1^), the Al─O bond (512 kJ mol^−1^) has the highest bond energy and thus is the most stable state (Figure 2e). After Al permeation, the Al‐O bond may enhance the orbital bonding capability between Al with O, thereby augmenting the chemical and structural stability of the NCM cathode materials.^[^ ^40^ ^]^ Furthermore, different metal‐O bond lengths under different chemical states were analyzed. After Al substitution, the bond length of Al─O becomes shorter (compared to the Co─O bond length). After the formation of oxygen defects, the bond length of Al─O octahedra becomes shorter, indicating that the invasion of Al can effectively suppress the destabilization and separation of lattice oxygen (Figure 2f).

The XRD refinements of Figure 2g,h suggest that, at an Al added amount of 5wt.%, the diffraction peaks of the NCM phase in the NCM‐Al mixed samples (0 and 800 rpm) remain consistent with the spinel structure of LiNiO_2_. The calculation results show that the crystal axis lengths *a*, *b*, and *c* as well as the unit cell volume of the NCM‐Al sample slightly increased (Table S1, Supporting Information). A crystal field theory diagram of the Co─O─Al bond was drawn (Figure 2i). In the bonding process between Co and O, the *π* electrons from O are repelled from the vacant 3*d* orbitals of Co atoms due to electrostatic repulsion. Conversely, Al and O atoms can form *sp* ^3^ *d* ^2^ hybridization to allow the *π* electrons of oxygen to occupy the vacant 3*d* orbitals and form a coordination bond. As a result, the Al─O bond exhibits a higher bond strength compared to the Co─O bond. These results confirm that Al impurities may bind with lattice O in NCM, stabilizing the crystal structures of the NCM cathode materials.

### 2.3 X‐ray Absorption Fine Structure Analysis of Co Sites in NCM‐Al Crystals

**Figure 3:**
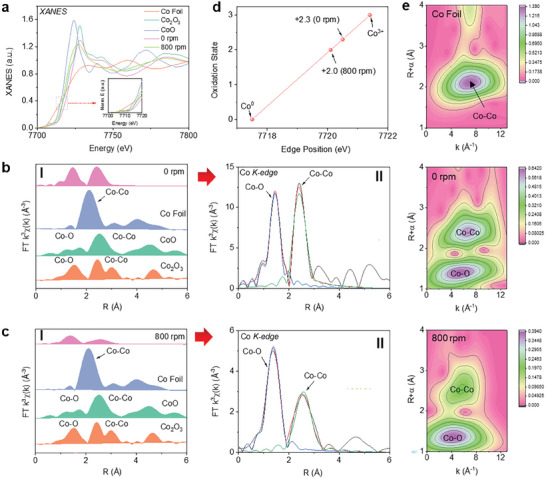
XANES and EXAFS examination of Co sites. a) Co *K*‐edge XANES of different NCM‐Al samples and reference Co foil, CoO, and Co_2_O_3_ samples. b) FT‐EXAFS of reference samples and the 0 rpm sample, with a red arrow pointing to the *R*‐space EXAFS fitting spectra of Co *K* edge at 0 rpm. c) FT‐EXAFS of reference samples and 800 rpm‐sample, with a red arrow pointing to the *R*‐space EXAFS fitting spectra of Co *K*‐edge at 800 rpm. d) Calculation results of Co chemical state in NCM‐Al and reference samples. e) WT‐EXAFS of Co foil and Co species in NCM‐Al samples using Co *K*‐edge *k* ^3^ weighted wavelet transform.

**Figure** **3**a presents the normalized Co K‐edge XANES spectra of the NCM‐Al‐0 sample, NCM‐Al‐800 sample, and Co, CoO, and Co_2_O_3_ reference samples, with an inset showing the enlarged absorption edge region. Owing to the presence of Ni/Co/Mn elements in NCM samples and the inability of X‐ray absorption spectra to distinguish adjacent coordination elements in the periodic table, Co─Co may also represent Co─Ni/Co/Mn. Compared to the NCM‐Al‐0 sample, the absorption edge of the NCM‐Al‐800 sample shifts towards lower energy, indicating a change in the local coordination environment of Co atoms, suggesting that penetrated Al acquires some electrons from Co. In the Co K‐edge XANES spectra, the absorption threshold of 800 samples lies between that of Co foil and Co─O, implying a potential reduction in the chemical valence state of Co following the Al permeation.

The bond lengths and metal coordination numbers were extracted from the Co K‐edge extended X‐ray absorption fine structure (EXAFS) fitting curve (Figure 3b,c). Analyses of the Co K‐edge Fourier transform *k* ^3^‐weighted EXAFS spectra and EXAFS wavelet transform indicate a significant decrease in relative peak intensity for the NCM‐Al‐800 sample compared to the NCM‐Al‐0 sample. Table S2 (Supporting Information) summarizes the EXAFS fitting results (coordination numbers and lengths of Co─O and Co─Co). The coordination numbers for Co─O and Co─Co (Ni or Mn) in the 0 sample were found to be 6.1 ± 0.7 and 7.1 ± 1.1, respectively, whereas following the Al permeation into NCM, the coordination numbers for Co─O and Co─Co in the 800‐rpm sample were determined to be 5.0 ± 0.7 and 6.0 ± 1.8, respectively. These coordination results confirm that Al permeation affects the local environment and electronic structure of transition metals by occupying Co─O and Co─Co coordination sites. The bond lengths for Co─O and Co─Co in the 0‐rpm sample were measured to be 1.90 ± 0.01 and 2.84 ± 0.01 Å, respectively, while after the Al occupancy of adjacent positions to Co, the bond lengths increased to 1.93 ± 0.01 and 2.94 ± 0.01 Å for Co─O and Co─Co, respectively. These findings are consistent with DFT calculations (Figure 2f), confirming that the Al permeation influences the coordination of Co─O and Co─Co and increases their bond lengths.^[^ ^41^ ^]^ Despite a significant difference in the Debye–Waller factor (σ^2^) between 0‐rpm and 800‐rpm samples, only slight increases in the coordination numbers of Co─O and Co─Co bonds were observed. Further analysis of the trend in the chemical valence state of Co species based on the EXAFS fitting results revealed a chemical valence state of +2.3 for NCM‐Al‐0, which decreased to +2.0 after Al permeation and reduction in NCM‐Al‐800 (Figure 3d). The wavelet transform (WT) analysis was performed to study the Co K‐edge EXAFS oscillations (Figure 3e). The Co K‐edge WT contour maps for the 0‐rpm and 800‐rpm samples exhibited similar trends, contrasting sharply with those of the Co foil.

### 2.4 Modelling and DFT Calculations of Al─O Vacancies in NCM Crystals

**Figure 4:**
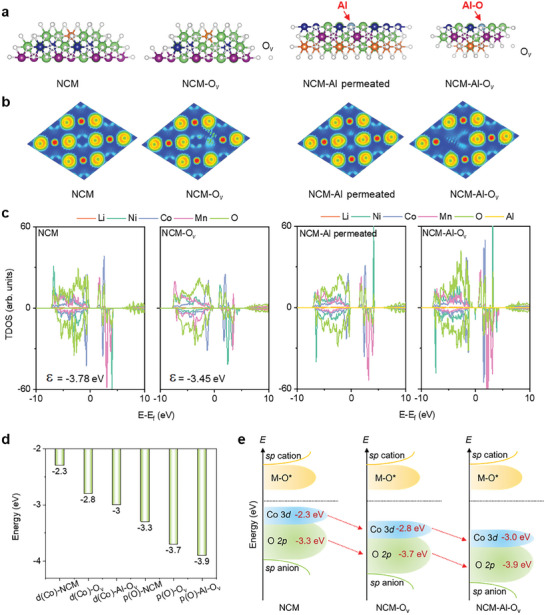
DFT analysis of Al permeation into NCM phase. a) Electronic configurations of NCM, NCM‐O_v_, NCM‐Al permeated, and NCM‐Al‐O_v_. (green = Li atoms, white = O atoms, blue = Co atoms, orange = Ni atoms, purple = Mn atoms, and light blue = Al atoms; view along with the c^*^ axis). b) ELF. c) DOS. d) Calculation results of the *d*‐band center of Co and *p*‐band center of O, and e) schematic diagram of the *d*‐band center of Co*3d* and *p*‐band center of O*2p*. NCM is the original sample, NCM‐O_v_ is the sample with oxygen defects, NCM‐Al permeated is the sample after Al replaces Co, and NCM‐Al‐O_v_ is the Al‐permeated sample with oxygen defects.

**Figure** **4**a presents the electron configurations of different NCM‐Al crystal structures, where NCM represents the original NCM cathode materials. The DFT calculation process is provided in Note S2 (Supporting Information). The sample after removing one O atom to form an O vacancy is denoted as NCM‐O_v_. The sample in which Al completely replaces Co is denoted as NCM‐Al permeated. The crystal configuration obtained by adding Al atoms into the NCM‐O_v_ sample is denoted as NCM‐Al‐O_v_. The electron localization functions (ELF) were calculated for NCM, NCM‐O_v_, NCM‐Al permeated, and NCM‐Al‐O_v_ samples (Figure 4b). In the NCM crystals, partially delocalized electrons persist around the Co atoms along the non‐bonding axis. Replacing Co by Al (NCM‐Al permeated sample) reduces the number of delocalized electrons, indicating strong covalent interactions between Al and O. After the generation of oxygen vacancies, a significant number of delocalized electrons are observed near the vacancy in NCM‐O_v_. Replacing Co by Al in NCM‐O_v_ can significantly alter the distribution of delocalized electrons in NCM‐Al‐O_v_.

A comparison of the density of states (DOS) for NCM and NCM‐O_v_ (Figure 4c) reveals that the introduction of oxygen vacancies in the recycling process causes the *d*‐band center of NCM‐O_v_ to shift upwards from −3.78 to −3.45 eV, closer to the Fermi level (for neutral oxygen vacancies). This leads to a decrease in the bandgap between the top and bottom of the valence band, which is beneficial for the separation of lattice oxygen, consistent with the ELF analysis results (Figure 4c). Conversely, by replacing Co with Al (NCM‐Al permeated), compared to the DOS results for NCM, the strong interaction of Al─O causes a downward shift of the Fermi level, promoting the stabilization of lattice oxygen in the NCM cathode materials. After oxygen vacancies (NCM‐Al‐O_v_) are formed, defect states are generated near Ni (in proximity to Al), crossing the Fermi level, and imparting a certain extent of metallic properties to the NCM crystalline materials. Furthermore, the *d*‐band center of Co and the *p*‐band center of O were calculated for NCM, NCM‐O_v_, and NCM‐Al‐O_v_ samples, respectively (Figure 4d). The *d*‐band center energy of NCM‐Al‐O_v_ is the lowest, and it is the furthest from the Fermi level. According to the *d*‐band center theory, the *d*‐band center further from the Fermi level exhibits lower reactivity. In contrast, the *d*‐band center of NCM is the closest to the Fermi level, indicating the highest chemical activity and thus the dissolution rate of NCM may be the fastest. The results for the *p*‐band center of O indicate that the average value (eV) of the *p*‐band center of NCM‐Al‐O_v_ decreases, making oxygen precipitation less likely, whereas the *p*‐band center of O in NCM is closer to the Fermi level, indicating weaker system stability compared to NCM‐Al‐O_v_ (Figure 4e). Thus, the permeation of Al into NCM crystals enhances the structural stability of the NCM crystals. This may lead to variable extent of reduction in the decomposition rate of NCM in different chemical reaction scenarios.

### 2.5 Significance of Aluminum Impurities in NCM Dissolution

**Figure 5:**
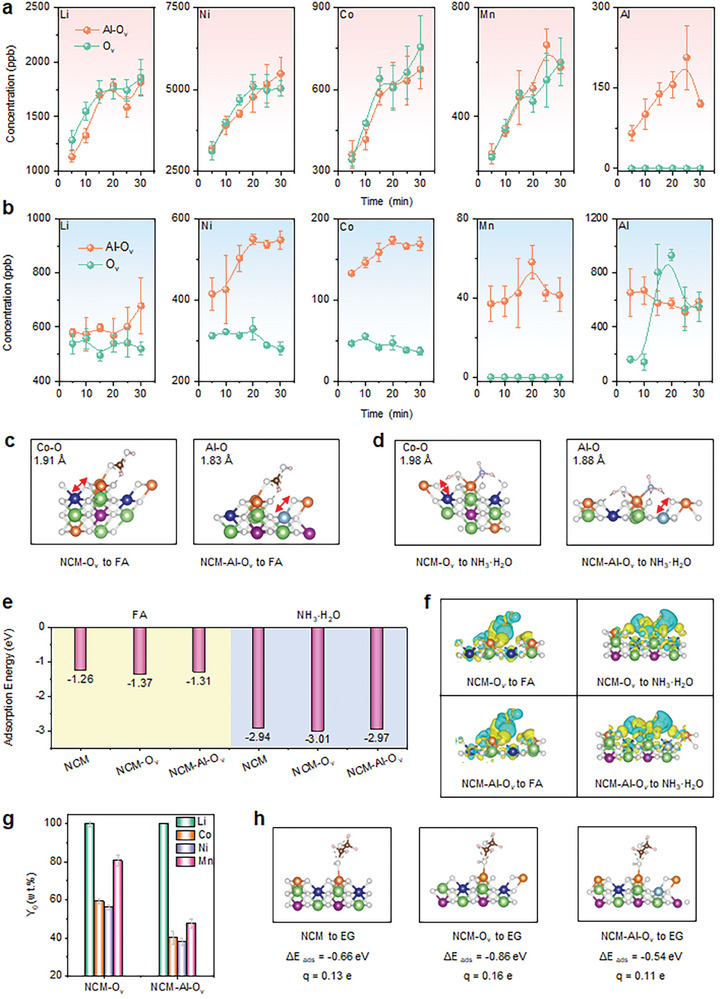
Dissolution of various NCM‐Al samples. Leaching kinetics of NCM‐O_v_ and NCM‐Al‐O_v_ in a) 5% (*v/v*) FA solution, b) 5% (*v/v*) NH_3_·H_2_O solution. Electronic adsorption configurations of NCM‐O_v_ and NCM‐Al‐O_v_ for c) FA and d) NH_3_·H_2_O molecules based on ab initio molecular dynamics and radial distribution function (view along the *a* axis); e) adsorption energy, and f) differential charge results (yellow denotes the accumulation of charges and blue, the depletion of charges) of FA and NH_3_·H_2_O molecules with different adsorption configurations (NCM, NCM‐O_v_, and NCM‐Al‐O_v_). g) metal leaching percentages of NCM‐O_v_ and NCM‐Al‐O_v_ in deep eutectic solvent (ChCl‐EG molar ratio of 2:1, 90 °C, and 1 h). h) adsorption energy (view along the *a* axis) and Bader charge calculation results of NCM, NCM‐O_v_, and NCM‐Al‐O_v_ on EG (green = Li atoms, white = O atoms, blue = Co atoms, orange = Ni atoms, purple = Mn atoms, brown = C atoms, and light blue = Al atoms).

In the formic acid (FA) solution, the dissolution patterns of Li, Ni, Co, and Mn exhibited similar trends (**Figure** **5**a). According to the E_h_‐pH curves, FA (pH of 2.1) acts as a proton hydrogen donor and has a reducing functional group, which can quickly react with lattice oxygen in NCM, leading to its dissolution and liberating Ni, Co, and Mn ions into the liquid phase environment (Figure S10a–d, Supporting Information). The leaching rates of transition metals in NCM‐O_v_‐Al were lower than those in NCM‐O_v_, confirming the inhibitory effect of Al on NCM dissolution, whereas the dissolution concentration of Al in NCM‐Al‐O_v_ was higher than that in NCM‐O_v_. The E_h_‐pH curve confirms that Al can exist as free ions in the range of pH 1–3 and exist in an ionic state under wide potential conditions (Figure S10e, Supporting Information).

In contrast, the dissolution behavior of NCM‐O_v_ and NCM‐Al‐O_v_ samples in NH_3_·H_2_O solution revealed similar dissolution trends for Al in both sets of samples, while it is important to note that Ni, Co, and Mn showed distinctively different trends (Figure 5b). Compared to NCM‐O_v_, NCM‐Al‐O_v_ exhibited higher dissolution concentrations of technology‐critical metal in NH_3_·H_2_O solution. According to the E_h_‐pH curve, Li exists as a free Li^+^ form, while Ni and Co combine with NH_4_⁺ to form soluble ligands in the liquid phase (Figures S11, Supporting Information). In Figure 5b, Mn from NCM‐Al‐O_v_ sample shows high solubility, while Mn from NCM‐O_v_ is hardly leached. As Mn usually exists as precipitated MnOOH and Mn(OH)_2_ in the NH_3_·H_2_O solution (Figure S11d, Supporting Information), therefore, the high solubility of Mn in the NCM‐Al‐O_v_ sample is likely attributed to the reduction of MnO_2_ (Figure 2c) and dissolution of low‐valence Mn species (Mn^0^, Mn^2+^, and Mn^3+^) in the liquid phase. The dissolution of Al reached equilibrium after 60 min, possibly in the form of metal‐aluminate ions (Figure 5b; Figure S11e, Supporting Information).

We performed ab initio molecular dynamics (AIMD) to visually demonstrate the impact of Al permeation on molecular bonds in NCM. The AIMD calculation process is provided in Note S3 (Supporting Information). The average Al─O bond length of NCM‐Al‐O_v_ in the FA adsorption system is ≈1.83 Å, whereas the Co─O bond length in NCM‐O_v_ is ≈1.91 Å (Figure 5c). The average Al─O bond length of NCM‐Al‐O_v_ in the NH_3_·H_2_O solution system is ≈1.88 Å, whereas the Co─O bond length in NCM‐O_v_ is ≈1.98 Å (Figure 5d). Figure 5e presents the electronic adsorption configurations of NCM, NCM‐O_v_, and NCM‐Al‐O_v_ for FA and NH_3_·H_2_O based on the DFT calculation. In the FA adsorption energy, the result for NCM‐Al‐O_v_ is −1.31 eV, while the values for NCM‐O_v_ and NCM are −1.37 and −1.26 eV, respectively. In the calculation results of the NH_3_·H_2_O system, the adsorption energy for NCM‐Al‐O_v_ is −2.97 eV, while the values for NCM‐O_v_ and NCM are −3.01 and −2.94 eV, respectively. Therefore, the formation of O vacancies can enhance the NCM reactivity, while Al permeation interferes with the separation and subsequent precipitation of lattice oxygen in different liquid‐phase environments.

Differential charge results (Figure 5f) indicate that compared to the FA system, the NH_3_·H_2_O system is more prone to charge transfer and exhibits higher chemical activity, consistent with the observed experimental results. The contrasting dissolution behavior of NCM‐Al samples in FA and NH_3_·H_2_O systems may originate from different chemical binding mechanisms. In the FA system, hydrogen proton released by FA molecules attacks the Al─O bond and (Ni/Co/Mn)─O bond, leading to the decomposition of the NCM crystals. In the NH_3_·H_2_O system, both O and N in NH_3_·H_2_O have lone pairs of electrons, which can effectively combine with metal ions and form stable soluble ligands. Therefore, the inhibitory effect of the Al─O bond on NH_3_·H_2_O molecule adsorption is far less than that of FA molecules.

We further examined the extraction of NCM‐O_v_ and NCM‐Al‐O_v_ in choline chloride‐ethylene glycol (ChCl‐EG, characteristics in Figure S12, Supporting Information) deep eutectic solvent (Figure 5g). The dissolution percentages of Ni, Co, and Mn in NCM‐Al‐O_v_ were significantly lower compared to NCM‐O_v_, while the leaching of Li remained relatively unaffected. Additional calculations were performed on the adsorption energies of ChCl‐EG with various electronic configurations (NCM, NCM‐O_v_, and NCM‐Al‐O_v_). The adsorption energy of NCM is ΔE_ads_ = −0.66 eV, and after the formation of oxygen vacancies in the recycling process, the adsorption energy of NCM‐O_v_ increased to −0.86 eV. This suggests that the presence of O_v_ can accelerate the process of NCM dissolution in ChCl‐EG. However, the adsorption energy of NCM‐Al‐O_v_ notably decreased to −0.54 eV (Figure 5h). The Bader charge confirms that the permeation of Al inhibits the transfer of electrons in the NCM surface and accordingly hinders the formation of oxygen vacancies within the lattice that is conducive to the subsequent breakdown of NCM active crystals. Our results reveal that the often‐overlooked interference of Al impurities in NCM crystals has distinctive influences on the dissolution of Li and NCM in various extraction environments.

## 3 Conclusions

It is noteworthy that recycling spent LIBs constitutes a critical component for the sustainable growth of the global new energy vehicle industry. Understanding the impact of indigenous impurities on the recovery of active crystals in spent LIB cathode materials is essential for regenerating high‐purity products of critical metals, including Li, Ni, Co, and Mn. Among the impurities, Al is identified as the most probable to infiltrate the active crystals of NCM cathode materials during physical crushing and sieving. Our experimental‐computational findings reveal that Al permeation and transformation in the NCM cathode materials can inhibit or promote their dissolution in various liquid‐phase environments. These new and mechanistic insights can provide valuable guideposts for reinventing the sustainable material circularity of the electric vehicle industry.

## 4 Experimental Section

### Materials and Reagents

Spent LiNi_0.5_Co_0.2_Mn_0.3_O_2_ (NCM523) LIBs were sourced from Xiaopeng Electric Vehicle Company in Guangdong, China. To eliminate residual electricity, the spent LIBs were first discharged using a homemade lighting device. Subsequently, NCM batteries were manually disassembled and separated to obtain powdered cathode electrode material for experimental uses. Materials, such as Al foil, Cu foil, separator, and the sorted outer shell, were recycled for further use. To dilute and dissolve the reaction samples, ultrapure deionized water was used in this investigation. As dissolution reagents, hydrochloric acid (HCl, AR, 36—38%) and nitric acid (HNO_3_, AR, 68–70%) from Aladdin Reagent Co., Ltd. were used for the analysis of the powdered NCM cathode material content. Choline chloride, ChCl (C_5_H_14_ClNO, AR, 98%), ethylene glycol, EG (C_2_H_6_O_2_, AR, 99%), formic acid, FA (HCOOH, AR, 88%), ammonia solution (NH_3_·H_2_O, AR, 25–28%), and Al powder (SP, 99.9%) were purchased from Aladdin Reagent Co., Ltd.

### Contact Friction and Leaching Experiments

To model the permeation response of Al impurities on spent LIB cathode materials, 0.1000 g of deactivated cathode material was mixed with 5 wt.% Al powder and subjected to frictional contacts in a planetary ball mill device containing four zirconia tanks (DECO‐PBM‐AD‐0.4L, DECO Technology Development Co., Ltd., Changsha, China). The rotational speed was 800 rpm, and the contact time was 5 min. Various solution systems were set up to analyze the leaching characteristics of NCM and Al, both pre‐friction (0 rpm) and post‐friction (800 rpm). System 1 contained a 5% (*v/v*) FA solution. Formic acid was used to simulate the traditional hydrometallurgical process with acid leaching, yet it was noted that the kinetics and extent of leaching of NCM would depend on the strength of acidity. System 2 contained a 5% (*v/v*) NH_3_·H_2_O solution, and System 3 was a deep eutectic solvents system with a ChCl‐EG molar ratio of 1:2. The leaching reaction was conducted under experimental conditions with a solid‐liquid ratio of 1:500, a temperature of 90 °C, and a reaction time of 60 min. The leaching concentrations of Li, Ni, Co, Mn, and Al were plotted on the *y*‐axis, while the *x*‐axis represents the time parameter. The leaching system for FA and NH_3_·H_2_O had a sampling interval of 10 min.

### Metal Concentration Analysis

The metal leaching solution was digested using a temperature‐programmed method with HNO_3_ at 180 °C for 10 h. Once the digestion solution reached room temperature, it was poured into a 100 mL volumetric flask with a 5% volume ratio of HNO_3_. The metal concentrations in the solution were determined using inductively coupled plasma‐optical emission spectrometry (ICP‐OES, Agilent 5110, USA). Metal ions were typically found in solutions ranging from 0 to 10.0 ppm, with concentration gradients of 0, 0.1, 0.5, 1.0, 5.0, and 10.0 ppm. Three solution samples were collected for digestion and testing at each experimental time node, and only the average values were presented. The error bars indicated the standard deviations, which measure the degree of dispersion of data points relative to the mean.

### Characterization Methods

The particle size distribution (PSD) of samples before and after the frictional reaction was quantified utilizing a particle size analyzer (Mastersizer 3000, Malvern, UK). Crystal structures of the specimen were probed using an X‐ray diffraction analysis system (XRD; Rigaku SmartLab, Japan) with a 2θ scanning range of 10°–80°. The 40 kV and 80 mA Cu Kα radiation (λ = 1.5406 Å) was applied at a current density of 200 mA and 45 kV, with a rate of 5° min^−1^. The chemical states of elements on the surfaces of solid samples were analyzed utilizing high‐resolution XPS with Al Kα radiation (ESCALAB 250Xi spectrometer, U.S.A.). Fourier‐transform infrared spectroscopy (FT‐IR, Nicolet 6700 spectrometer, U.S.A.; range: 4000–400 cm^−1^) was employed with a total of 16 scans to analyze modifications in functional groups on the specimen. The morphology and microstructure of the solid samples were examined using field‐emission scanning electron microscopy (FESEM; Zeiss S‐3500N, Germany) and EDS mapping (X‐Max 20, Oxford instruments; Abingdon, UK). High‐resolution, aberration‐corrected, high‐angle annular dark field‐scanning transmission electron microscopy (AC HAADF‐STEM) imaging of the materials was conducted utilizing an FEI Tecnai F30 instrument (Thermo Fisher Scientific, USA). The X‐ray absorption spectra (XAS) of the samples at O K‐edge (538 eV) were obtained at the Singapore Synchrotron Light Source (SSLS) center using a pair of channel‐cut Si (111) crystals in the monochromator (detector: ion chamber), which also included the X‐ray absorption near‐edge structure (XANES) and extended X‐ray absorption fine structure (EXAFS) (Note S4, Supporting Information). Gibbs free energy calculation was performed using HSC Chemistry 8.0 software.

## Conflict of Interest

The authors declare no conflict of interest.

## Supporting information



Supporting Information

## Data Availability

The data that support the findings of this study are available in the supplementary material of this article.
