# Supplementary material for: Dissolution of Spent Lithium‐Ion Battery Cathode Materials: Overlooked Significance of Aluminum Impurities
Source: Adv Sci (Weinh). 2025 Feb 28;12(21):2417737. doi: 10.1002/advs.202417737 (PMC12140308; doi:10.1002/advs.202417737)
Supplement: Supplementary file 1 — Supporting Information [file ADVS-12-2417737-s001.docx]

**Supplementary Information**

**Dissolution of Spent Lithium-ion Battery Cathode Materials: Overlooked Significance of Aluminum Impurities**

Kang Liu, Yuying Zhang, Mengmeng Wang, Xiaohong Zhu, Roya Maboudian, Daniel C.W. Tsang*

K. Liu, Y. Zhang, M. Wang, D.C.W. Tsang

Department of Civil and Environmental Engineering, The Hong Kong University of Science and Technology, Clear Water Bay, Hong Kong, China

E-mail: cedan@ust.hk

X. Zhu

Department of Civil and Environmental Engineering, University of California Berkeley, Berkeley, California 94720, United States

Department of Chemical and Biomolecular Engineering, University of California Berkeley, Berkeley, California 94720, United States

R. Maboudian

Department of Chemical and Biomolecular Engineering, University of California Berkeley, Berkeley, California 94720, United States

Contents:

1. Figures S1-S12.

2. Tables S1-S2.

3. Notes S1-S4.

**Table of Contents**

Figure S1. XRD patterns of NCM-Al before and after frictional reaction. 3

Figure S2. SEM-Mapping results of NCM-Al samples: (a) (b) 0 rpm and (c) (d) 800 rpm. 4

Figure S3. Size distribution results: NCM, Al, NCM-Al-0 rpm, and NCM-Al-800 rpm. 5

Figure S4. Characterization of NCM-Al samples: (a) TEM, (b) SAED, (c) EDS mapping (800 rpm). 6

Figure S5. Gibbs free energy reaction pathway between Al and different oxides. 7

Figure S6. Phase diagram of Li-Al-O under different Al doping levels. 8

Figure S7. XPS high-resolution energy spectra results of Li*1s*. 9

Figure S8. Phase diagram of Me-Al-O under different Al doping levels. 10

Figure S9. FT-IR spectra of NCM, Al, NCM-Al 0 rpm, and NCM-Al 800 rpm. 11

Figure S10. E_h_-pH curve of (a) Li, (b) Ni, (c) Co, (d) Mn, and (e) Al-HCOOH-H_2_O. 12

Figure S11. E_h_-pH curve of (a) Li, (b) Ni, (c) Co, (d) Mn, and (e) Al-NH_3_-H_2_O. 13

Figure S12. (a) pKa and pKb values of ChCl-EG, ChCl, and EG. (b) configurations for LUMO and HUMO. (c) calculation results of LUMO and HUMO. 14

Table S1. EXAFS fitting parameters at the Co *K*-edge (*Ѕ*_0_^2^ = 0.68) 15

Table S2. Linear Combination fitting 17

Note S1 18

Note S2 19

Note S3 20

Note S4 21


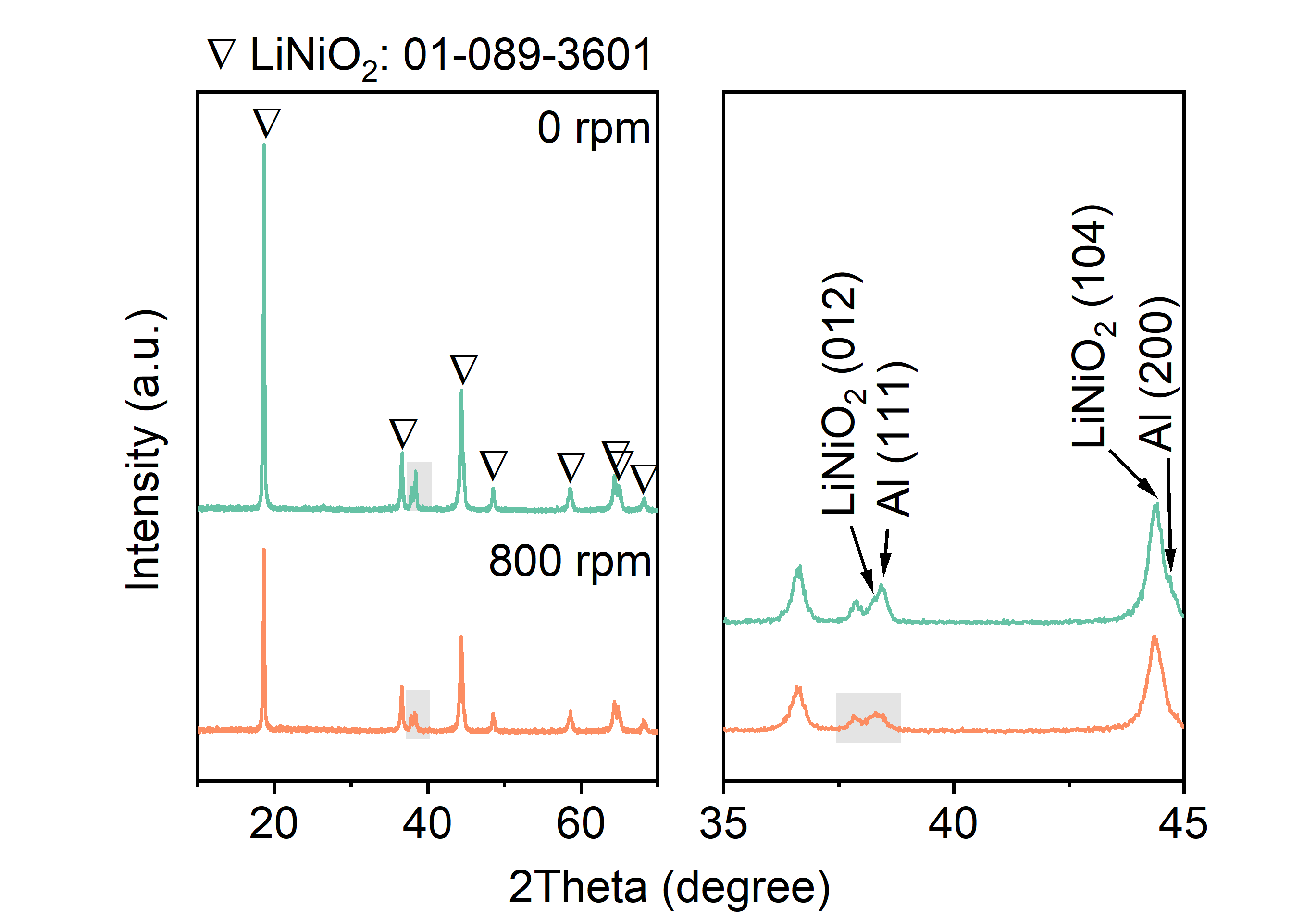


# Figure S1. XRD patterns of NCM-Al before and after frictional reaction.

As shown in Figure S1 (left), the characteristic diffraction peaks of LiNiO_2_ (JCPDF: 01-089-3601) can be seen in the XRD pattern, confirming that frictional reaction did not cause significant damage to NCM's spinel crystal structure, but only a reduction in the diffraction peaks. The enlarged pattern shows distinct diffraction peaks for LiNiO_2_ (012) and Al (111), as well as LiNiO_2_ (104) and Al (200), indicating that the 0-rpm sample is a mixture of NCM and Al (Figure S1, right).


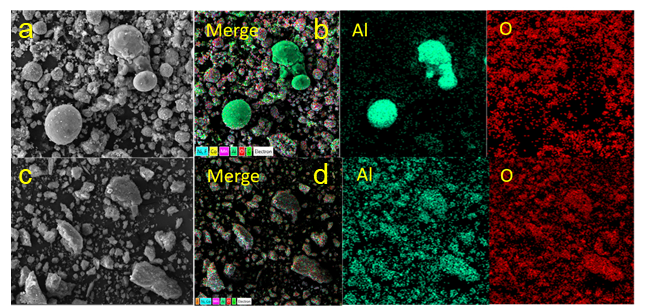


# Figure S2. SEM-Mapping results of NCM-Al samples: (a) (b) 0 rpm and (c) (d) 800 rpm.

Under Scanning Electron Microscope (SEM) with an Energy Dispersive Spectrometer (EDS) Mapping, circular Al particles and irregular NCM particles can be observed (Figure S2).


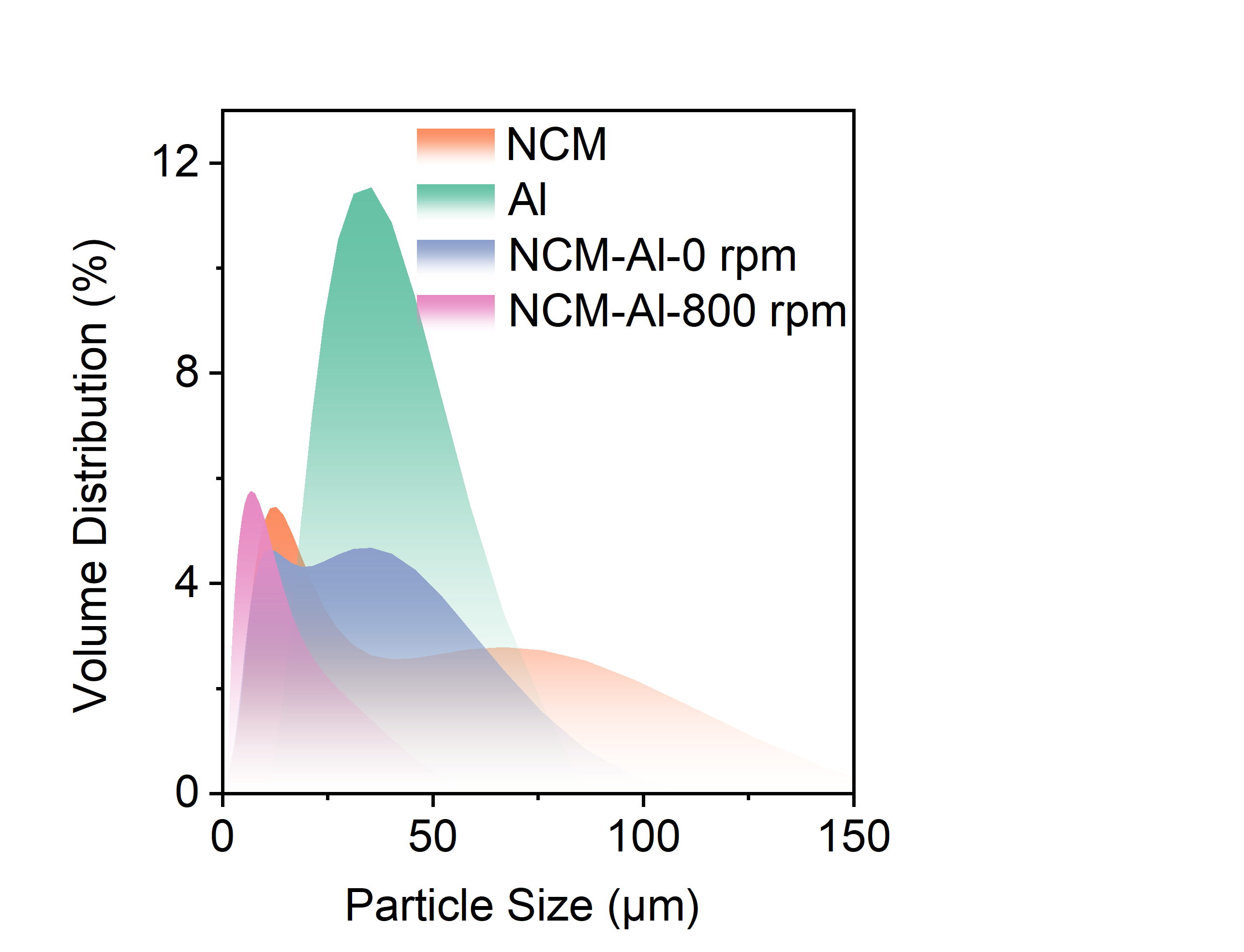


# Figure S3. Size distribution results: NCM, Al, NCM-Al-0 rpm, and NCM-Al-800 rpm.

After the friction reaction treatment, the Al particles exhibited a trend of pulverization, dispersing uniformly into the interior of the NCM particles. Laser particle size analyzer results confirmed the homogenization of NCM and Al powder after the friction treatment. The particle size decreased from 33 microns to 6 microns (Figure S3).


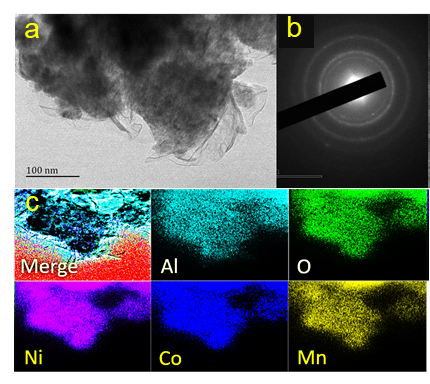


# Figure S4. Characterization of NCM-Al samples: (a) TEM, (b) SAED, (c) EDS mapping (800 rpm).

The High-Resolution Transmission Electron Microscope (HR-TEM) results of the NCM-Al sample are shown in Figure S4, where cloud-like structures and polycrystalline rings can be observed. In the EDS results, Al is uniformly distributed within the NCM.


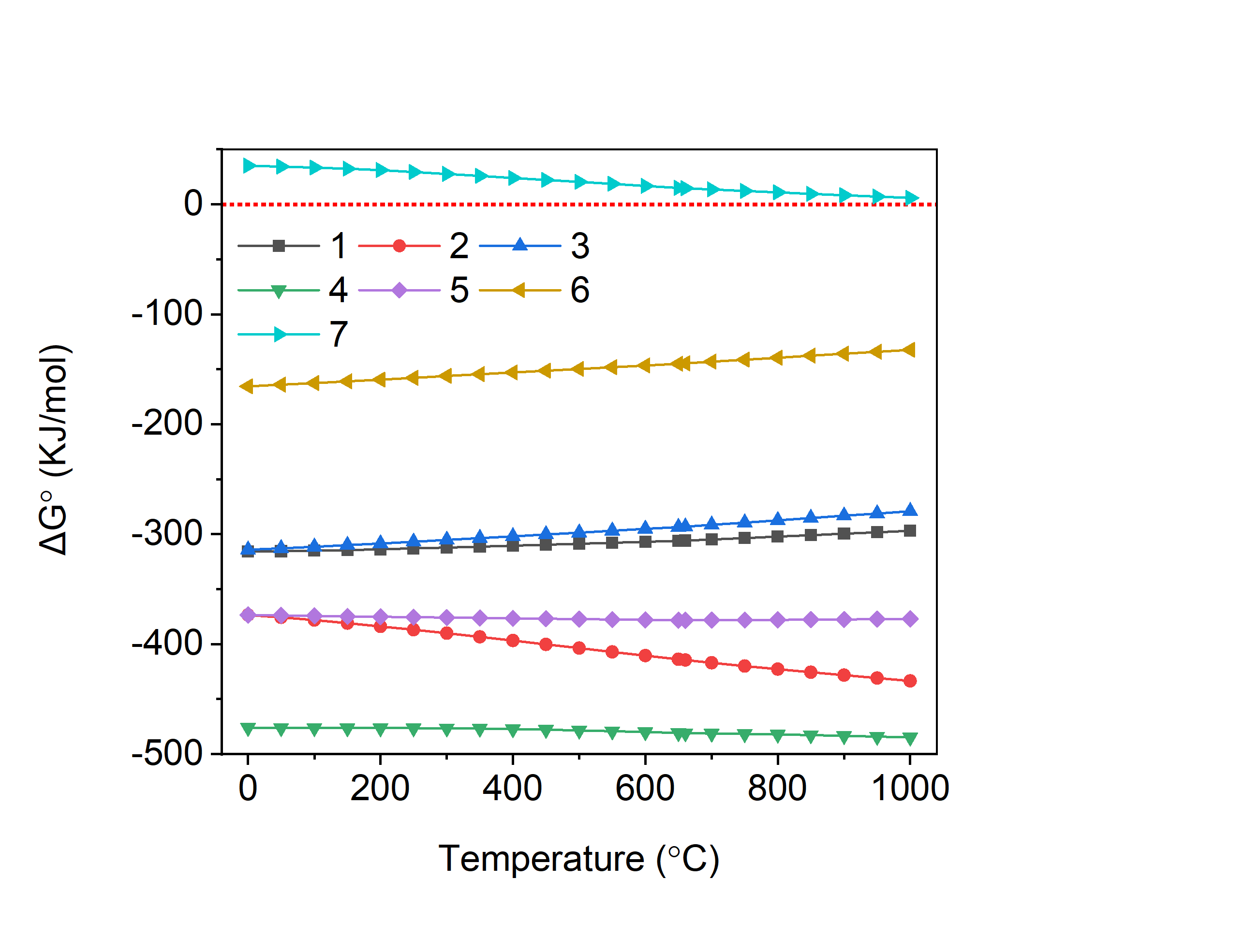


# Figure S5. Gibbs free energy reaction pathway between Al and different oxides.

| Eq. *n* | Chemical Formula |
| --- | --- |
| 1 | NiO + 2/3Al = Ni + 1/3Al_2_O_3_ |
| 2 | Co_3_O_4_ + 2/3Al = 3CoO + 1/3Al_2_O_3_ |
| 3 | CoO + 2/3Al = Co + 1/3Al_2_O_3_ |
| 4 | 2MnO_2_ + 2/3Al = Mn_2_O_3_ + 1/3Al_2_O_3_ |
| 5 | Mn_2_O_3_ + 2/3Al = 2MnO + 1/3Al_2_O_3_ |
| 6 | MnO + 2/3Al = Mn + 1/3Al_2_O_3_ |
| 7 | Li_2_O + 2/3Al = Li + 1/3Al_2_O_3_ |

The Gibbs free energy reaction pathway was analyzed to calculate the possible binding pathway between NCM and Al. The reaction pathways of Al with Li and different transition metal oxides were verified and shown in Figure S5. According to the Gibbs free energy change of the reduction reaction, the priority order of its occurrence is (Eq.4) > (Eq.2) > (Eq.5) > (Eq.1) > (Eq.3) > (Eq.6), that is, the order of the reaction products appearing is Mn_2_O_3_ → CoO → MnO → Ni → Co → Mn. Li will not be reduced by Al but exists in the form of LiAlO_2_.


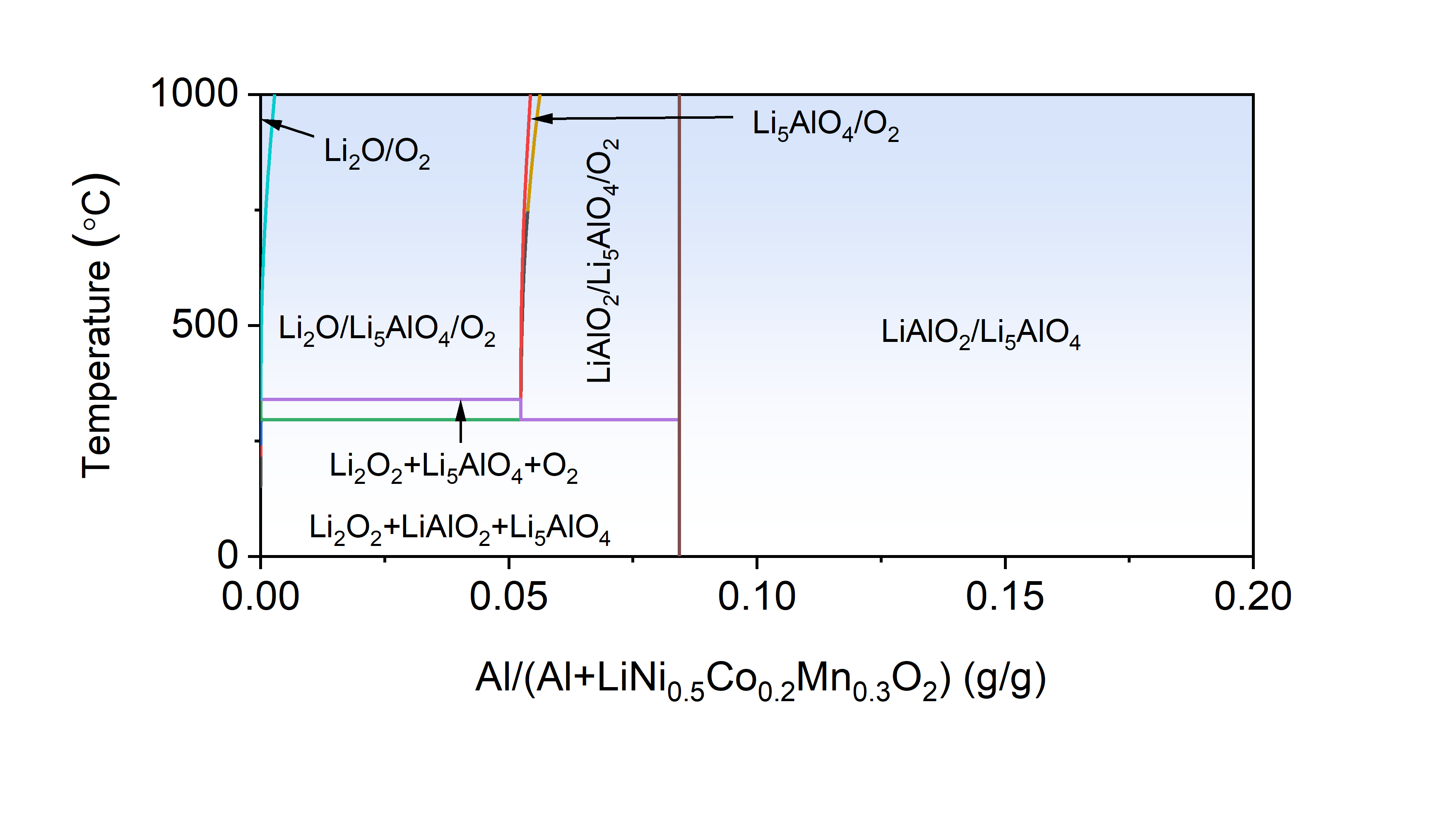


# Figure S6. Phase diagram of Li-Al-O under different Al doping levels.

Phase diagrams (calculated using FACTSage 6.0) were employed to investigate the potential phase composition of Al and NCM crystals after the friction reaction. At a doping level of 5 wt.% Al, it is believed that Al is prone to form Li_2_O_2_ + LiAlO_2_ + Li_5_AlO_4_ phases through facile binding with Li (Figure S6).


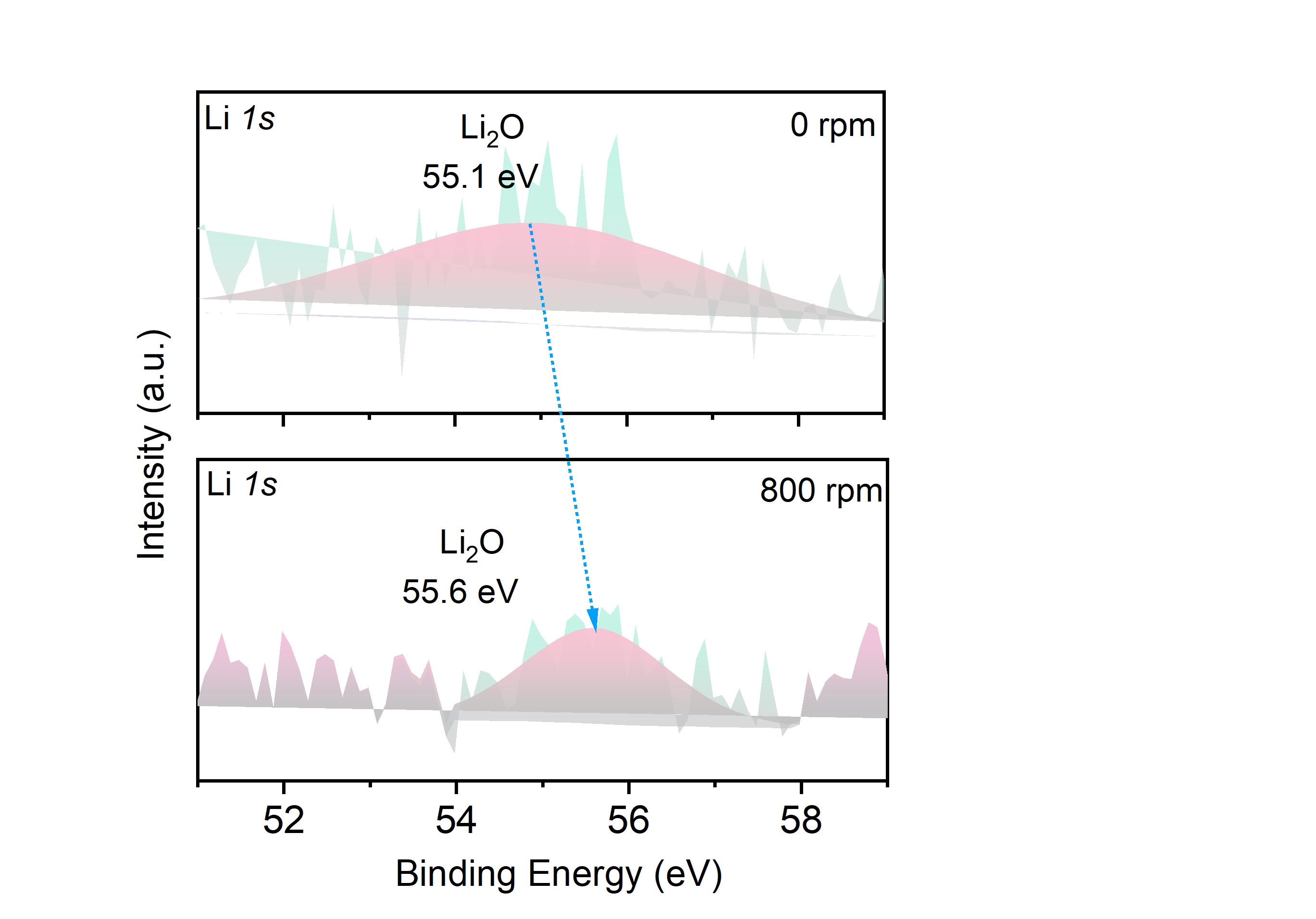


# Figure S7. XPS high-resolution energy spectra results of Li*1s*.

High-resolution spectra of Li*1s* show that Li still forms Li_2_O species before and after the friction reaction (Figure S7).


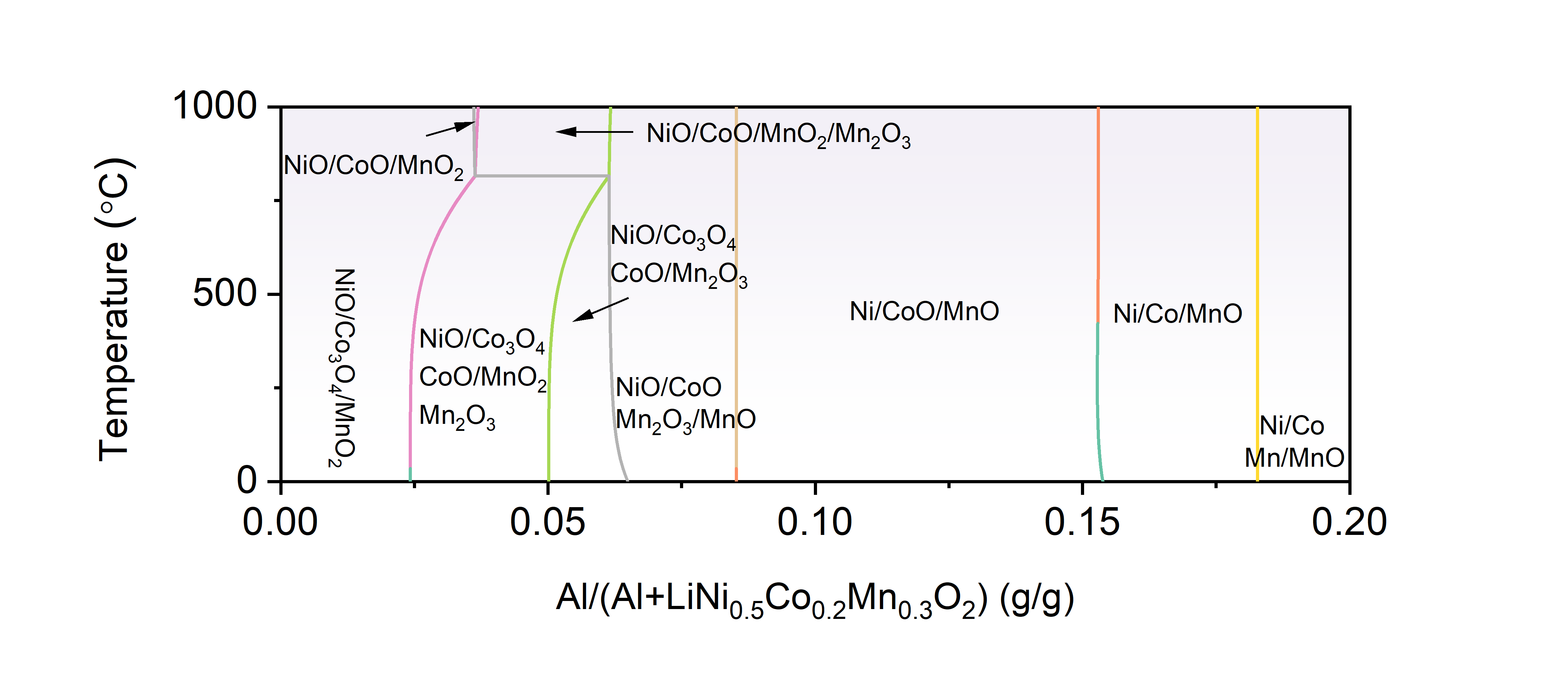


# Figure S8. Phase diagram of Me-Al-O under different Al doping levels.

(Me represents the Ni, Co, and Mn).

Transition metals such as Ni, Co, and Mn can be solid phase reduced by Al to form mixed valence oxides (Figure S8). In the phase diagram of the Al/LiNi_0.5_Co_0.2_Mn_0.3_O_2_ system, as the content of the reducing agent Al increases, the Ni, Co, and Mn elements in LiNi_0.5_Co_0.2_Mn_0.3_O_2_ are successively reduced. The order of the reduction reaction is consistent with the description of Gibbs free energy results, but the effect of temperature on whether the reduction reaction occurs is not significant.


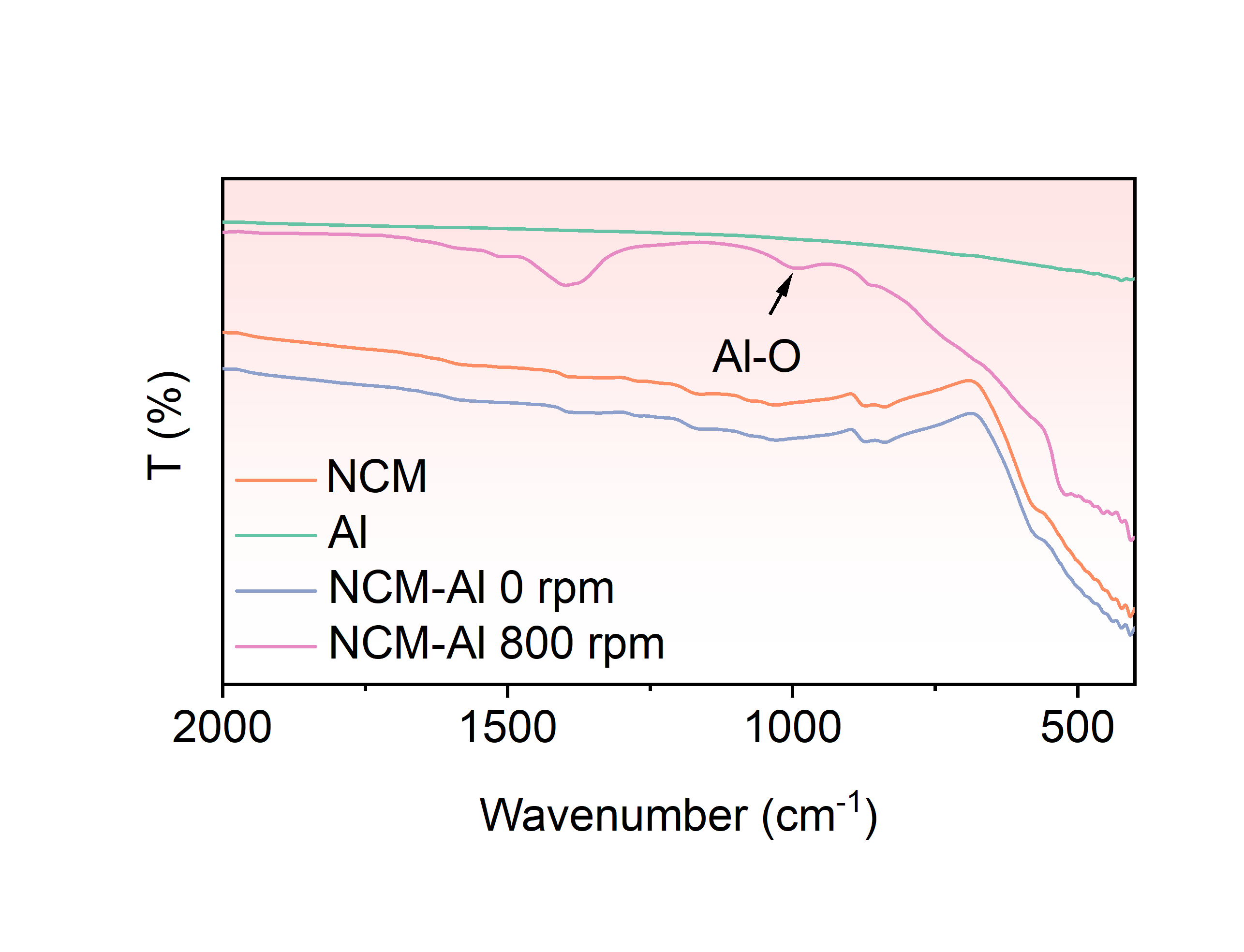


# Figure S9. FT-IR spectra of NCM, Al, NCM-Al 0 rpm, and NCM-Al 800 rpm.

In the detection based on Fourier transform infrared spectrometer testing, we also found the presence of Al-O bonds at 995.3 cm^-1^ of the 800-rpm sample, confirming the chemical binding of Al with lattice oxygen in NCM crystals (Figure S9).


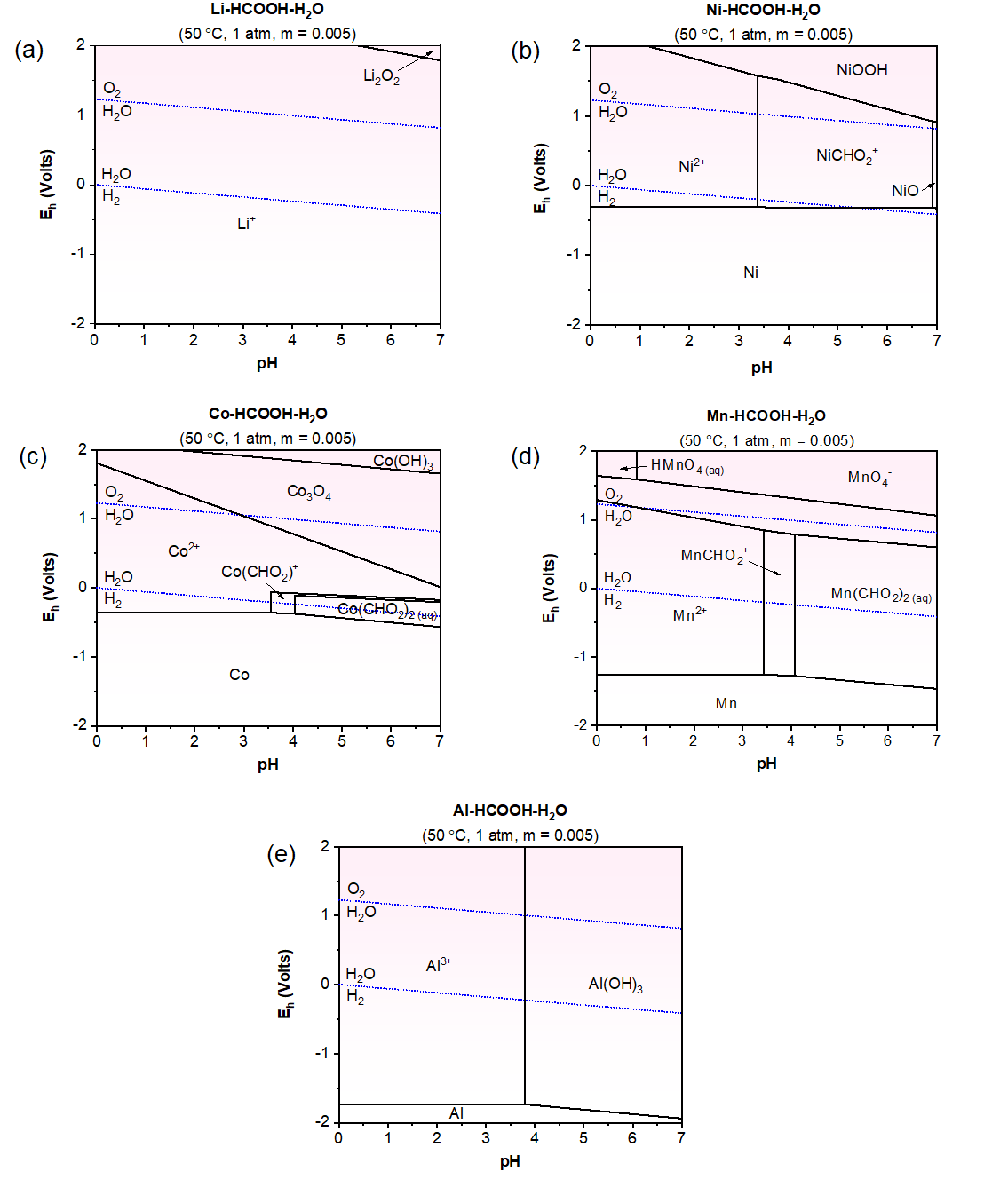


# Figure S10. E_h_-pH curve of (a) Li, (b) Ni, (c) Co, (d) Mn, and (e) Al-HCOOH-H_2_O.


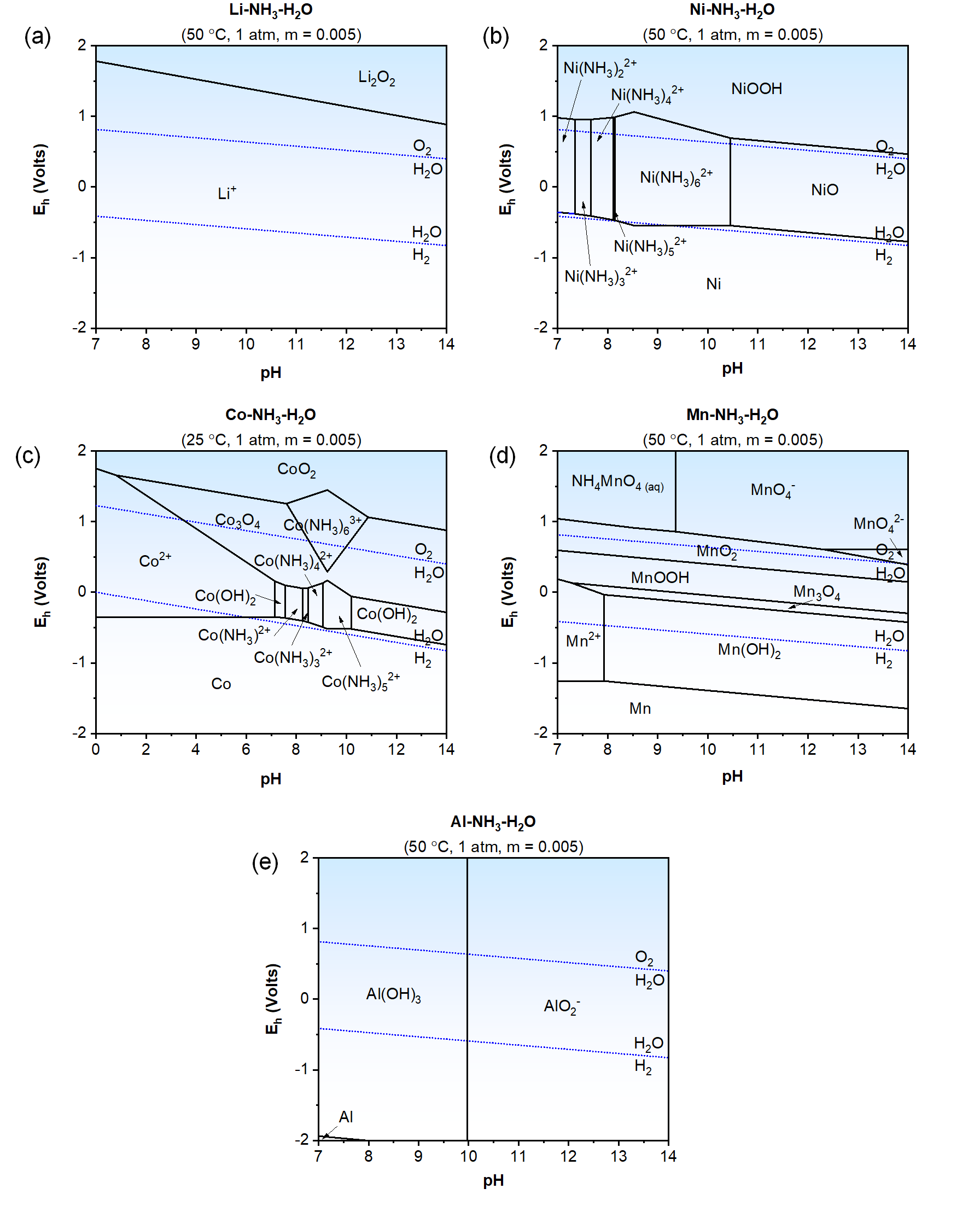


# Figure S11. E_h_-pH curve of (a) Li, (b) Ni, (c) Co, (d) Mn, and (e) Al-NH_3_-H_2_O.

**Figure S12.** (a) pKa and pKb values of ChCl-EG, ChCl, and EG. (b) configurations for LUMO and HUMO. (c) calculation results of LUMO and HUMO.

Deep eutectic solvents (DESs) are newly developed reaction systems used to extract critical metals from spent LIB cathode materials. This study delves deeper into examining the leaching characteristics of NCM and NCM-Al in DESs. The pKa and pKb values of choline chloride-ethylene glycol (ChCl-EG) DES, ChCl, and EG were calculated. ChCl showed alkalinity, while EG showed acidity (Fig. 12a). Once the hydrogen bond donor and acceptor are linked by hydrogen bonds to create a DES, the solvent's pH is approximately 8.67. The highest occupied molecular orbital (HOMO) and lowest unoccupied molecular orbital (LUMO) values of ChCl-EG were combined to determine their redox characteristics (Fig. 12b). The LUMO value of ChCl-EG is approximately -0.86 eV, indicating a decrease compared to ChCl (-0.92 eV) and a notable increase compared to EG (-0.14 eV) (Fig. 12c). This confirms that the reducibility of the ChCl-EG system is primarily due to ChCl. ChCl-EG can create an alkaline reduction system that enhances the dissolution of NCM and NCM-Al-O_v_.

**Table S1.** EXAFS fitting parameters at the Co *K*-edge (*Ѕ*_0_^2^ = 0.68)

| Sample | Path | C.N. | *R* (Å) | σ^2^×10^3^ (Å^2^) | *ΔE* (eV) | *R* factor % |
| --- | --- | --- | --- | --- | --- | --- |
| Co foil | Co-Co | 12* | 2.49 ± 0.01 | 5.6 ± 0.5 | -1.7 ± 0.7 | 0.002 |
| Co 800 rpm | Co-O | 5.0 ± 0.7 | 1.93 ± 0.01 | 8.3 ± 1.9 | -3.1 ± 1.7 | 0.010 |
|  | Co-Co | 6.0 ± 1.8 | 2.94 ± 0.02 | 15.6 ± 3.2 | 5.8 ± 2.1 |  |
| Co 0 rpm | Co-O | 6.1 ± 0.7 | 1.90 ± 0.01 | 2.8 ± 1.1 | -4.1 ± 1.7 | 0.005 |
|  | Co-Co | 7.1 ± 1.1 | 2.84 ± 0.01 | 5.7 ± 1.2 | -8.8 ± 1.6 |  |

*C.N.*: coordination numbers; *R*: bond distance; *σ*^2^: Debye-Waller factors; Δ*E*: the inner potential correction. *R* factor: goodness of fit.

* Fitting with fixed parameters.

Due to the presence of Ni/Co/Mn elements in the sample, and the inability of XAS to distinguish adjacent coordination elements in the periodic table, Co-Co in the above table can also be written as Co-Ni/Co/Mn, indicating that Co-Ni/Co/Mn cannot be distinguished or exists simultaneously.

Although the two samples only have different processing methods, there are significant differences in the data testing results, especially in terms of data quality. It is uncertain whether data quality has a significant impact on the analysis results (because it is not known if the data quality is good). From the analysis results, there is a difference in the average coordination number between the two samples, but the difference is not significant (considering errors); There is a certain difference in key length. In linear combination fitting component analysis, Co-0 rpm sample attempted to introduce CoO, but the weight corresponding to CoO was 0, so CoO was removed here. Of course, if CoO or +2 valent Co is expected to exist, but Co^0^ does not exist, then you can try removing Co foil. Due to the requirement document specifying the use of Co foil, CoO, and Co_2_O_3_ for fitting, only the fitting results of this combination are provided here.

**Table S2.** Linear Combination fitting

| Sample | Weight of standards | | | Valance | R factor |
| --- | --- | --- | --- | --- | --- |
|  | Co_2_O_3_ (+3) | CoO (+2) | Co foil (0) |  |  |
| Co-800 rpm | 0.47 ± 0.03 | 0.32 ± 0.03 | 0.21 ± 0.02 | + 2.0 | 0.005 |
| Co-0 rpm | 0.76 ± 0.03 | / | 0.24 ± 0.03 | + 2.3 | 0.020 |

**Note S1**

To achieve the doping of Al in NCM cathode materials, initially, a mass ratio of 5 wt.% Al powder was mixed with the disassembled NCM cathode materials and subjected to a friction reaction for 5 minutes. Under the influence of multi-component mechanical forces such as shear, friction, and impact, Al infiltrated the lattice of the active crystals in NCM cathode materials. Subsequently, the NCM-Al samples (0 rpm and 800 rpm) were characterized and applied to acidic (formic acid solution), alkaline (ammonia solution), and solvent (deep eutectic solvent, DES) reaction environments to investigate the changes in leaching behaviors of critical metals. During this process, the pre-friction-treated NCM-Al sample (0 rpm) served as a control (see detailed information in the methods section).

**Note S2**

Density functional theory calculations.

In this study, the Vienna Ab initio Simulation Package (VASP) software package was utilized for DFT calculations. Projection Enhanced Waves (PAW) were used to elucidate electron-nucleus interactions and associated exchange potentials. The exchange-correlation can be estimated using the Perdew Burke Ernzerhof (PBE) functional within the Generalized Gradient Approximation (GGA) framework. The Hubbard + U correction was applied to GGA to account for the Coulomb interactions between Ni, Co, and Mn atoms. The authors utilized U values of 3.9 eV for Ni, 3.32 eV for Co, and 6.2 eV for Mn, based on prior research. The plane wave energy cut-off value was 500 eV, and the force convergence standard was set to be below 0.03 eV/Å for all atoms. A 15 μm vacuum layer was applied in the z-direction to prevent potential interactions between periodic units. The Grime DFT-D3 method with zero damping function accounted for long-range van der Waals (vdW) interactions. When modeling surface adsorption, utilizing the NCM (010) surface as the adsorption site was advisable.

**Note S3**

*Ab initio molecular dynamics simulations.*

The structure simulated by ab initio molecular dynamics (AIMD) adopts the geometrically optimized configuration as the initial model. The CP2K program (version 2024.1) was used for dynamic calculations, employing the PBE functional and a hybrid Gaussian/Plane-Wave method (GPW). The conventional (NVT) ensemble was used to sample the simulations, employing Nose-Hoover thermostats with a time step of 0.5 *fs*. The simulations were conducted at a finite temperature of 300 K for a duration exceeding 5 *ps*. The valence electrons' molecular orbitals were extended into DZVP-MOLOPT-SR-GTH basis sets, whilst the atomic core electrons were characterized using Goedecker-Teter-Hutter pseudopotentials. At the energy cutoff of 500 Ry, the plane-wave basis set was shortened. To optimize the structures, a stringent self-consistent field threshold of 1E-7 arbitrary units was employed. The DFT-D3 method was used to apply dispersion correction in all calculations. The dipole correction technique was employed consistently to mitigate the occurrence of spurious charge interactions between adjacent images inside the periodic boundary condition.

**Note S4**

Fitting of synchrotron radiation data.

The transmission mode was used to record the O K-edge XANES data. With an average electron current of less than 200 mA, the storage ring operated at an energy of 700 MeV. The XANES data were recorded in a transmission mode for the Co K-edge. As references, Co foil, Co_3_O_4_, and CoO were utilized. With an average electron current of less than 200 mA, the storage ring operated at 2.5 GeV of energy. The ATHENA module included in the IFEFFIT software packages was used to extract and analyze the obtained EXAFS data in accordance with conventional protocols. The obtained EXAFS data were processed in Athena (version 0.9.26) for background, pre-edge line and post-edge line calibrations. Then Fourier transformed fitting was carried out in Artemis (version 0.9.26). The *k*^3^ weighting, *k*-range of 2 - ~11 Å^-1^ and R range of 1 - 3 Å were used for the fitting. The four parameters, coordination number, bond length, Debye-Waller factor and E_0_ shift (CN, R, σ^2^, ΔE_0_), were fitted without any parameter being fixed, constrained, or correlated. For Wavelet Transform analysis, the χ(*k*) exported from Athena was imported into the Hama Fortran code. The parameters were listed as follows: R range, 1 - 4 Å, *k* range, 0 - ~11 Å^-1^; *k* weight, 2; and Morlet function with κ = 10, σ = 1 was used as the mother wavelet to provide the overall distribution.
